# Toward a Circular Bioeconomy: Development of Pineapple Stem Starch Composite as a Plastic-Sheet Substitute for Single-Use Applications

**DOI:** 10.3390/polym15102388

**Published:** 2023-05-19

**Authors:** Chanaporn Thongphang, Atitiya Namphonsane, Sombat Thanawan, Chin Hua Chia, Rungtiwa Wongsagonsup, Siwaporn Meejoo Smith, Taweechai Amornsakchai

**Affiliations:** 1 Center of Sustainable Energy and Green Materials, Faculty of Science, Mahidol University, Phuttamonthon 4 Road, Salaya, Nakhon Pathom 73170, Thailandsiwaporn.smi@mahidol.ac.th (S.M.S.); 2 Rubber Technology Research Center, Faculty of Science, Mahidol University, Phuttamonthon 4 Road, Salaya, Nakhon Pathom 73170, Thailand; sombat.tha@mahidol.ac.th; 3 Department of Applied Physics, Faculty of Science and Technology, Universiti Kebangsaan Malaysia, Bangi 43600, Selangor, Malaysia; chia@ukm.edu.my; 4 Division of Food Technology, Kanchanaburi Campus, Mahidol University, Kanchanaburi 71150, Thailand; rungtiwa.won@mahidol.ac.th; 5 Food and Nutrition Academic and Research Cluster, Institute of Nutrition, Mahidol University, Nakhon Pathom 73170, Thailand

**Keywords:** biodegradable plastic, starch, circular economy, pineapple, tensile strength

## Abstract

Plastic waste poses a significant challenge for the environment, particularly smaller plastic products that are often difficult to recycle or collect. In this study, we developed a fully biodegradable composite material from pineapple field waste that is suitable for small-sized plastic products that are difficult to recycle, such as bread clips. We utilized starch from waste pineapple stems, which is high in amylose content, as the matrix, and added glycerol and calcium carbonate as the plasticizer and filler, respectively, to improve the material’s moldability and hardness. We varied the amounts of glycerol (20–50% by weight) and calcium carbonate (0–30 wt.%) to produce composite samples with a wide range of mechanical properties. The tensile moduli were in the range of 45–1100 MPa, with tensile strengths of 2–17 MPa and an elongation at break of 10–50%. The resulting materials exhibited good water resistance and had lower water absorption (~30–60%) than other types of starch-based materials. Soil burial tests showed that the material completely disintegrated into particles smaller than 1 mm within 14 days. We also created a bread clip prototype to test the material’s ability to hold a filled bag tightly. The obtained results demonstrate the potential of using pineapple stem starch as a sustainable alternative to petroleum-based and biobased synthetic materials in small-sized plastic products while promoting a circular bioeconomy.

## 1. Introduction

In recent years, there has been growing concern about the environmental impact of plastics, which are known to persist in the environment and harm living organisms [1]. To address this issue, a variety of solutions have been proposed, including the use of biodegradable polymers in single-use applications and outright bans on plastic use in some countries [2]. Biodegradable polymers are available in a variety of forms, including fully biobased polylactic acid (PLA), partially biobased polybutylene succinate (PBS), fully synthetic polybutylene adipate terephthalate (PBAT), and natural polymers such as starch, polyhydroxyalkanoates (PHAs), and polyhydroxybutyrate (PHB). However, not all biodegradable polymers are created equal, and it is important to understand the specific properties and limitations of each type. For example, polylactic acid (PLA), polybutylene adipate terephthalate (PBAT), and polybutylene succinate (PBS) are widely used biodegradable polymers, but they do not easily biodegrade in natural environments. Instead, they require specific conditions, such as controlled humidity and temperature, which are typically only found in industrial composting facilities [3,4]. Therefore, it is still crucial to ensure that these biodegradable materials are properly disposed of and collected in such facilities to ensure their complete degradation. On the other hand, starch-based materials, polyhydroxyalkanoates (PHAs), and polyhydroxybutyrates (PHBs) are fully biodegradable in natural environments and may be a more appropriate choice for certain applications where collection and recycling are not easy or economical, such as small and lightweight objects or products. These materials can biodegrade completely without requiring specialized industrial composting facilities and may, therefore, offer a more practical and environmentally friendly solution for certain types of waste.

Considering the availability, cost of production, and other environmental impacts, starch may be the material of choice to make bioplastics and, indeed, there are many reviews available [5,6,7]. However, unmodified starch has poor properties, especially low mechanical strength and poor water resistance. Thus, it requires modification or blending with other polymers to make it more useful [8,9,10,11]. Unfortunately, most starches are used for human consumption. Using them for materials would certainly interrupt food supply chains and limit access to food for vulnerable groups, making it unsustainable. Researchers have investigated various nonconventional or nonfood starches for use in material applications [12,13,14,15,16,17,18]. However, the availability of these alternative starch sources is relatively limited. As a result, much of the research on starch-based materials has focused on modifying traditional food-grade starch to improve its properties. One of the main challenges of using starch as a material is its poor water resistance and low mechanical strength. To address these limitations, researchers have developed a range of modification methods. Initially, modification was achieved through a single method, but the resulting products still had limitations. Over time, researchers developed more advanced modification techniques, such as dual modifications [19,20,21,22] or combining starch with various fillers [23,24,25]. This not only adds complexity to the process but also produces products with higher intensity in terms of material and energy, leading to a higher carbon footprint. Therefore, finding starch that does not require modification or requires very little modification would make it more sustainable.

Recently, our group started investigating pineapple stem starch (PSS) as a promising material for a variety of applications. This is because the PSS has a relatively high amylose content and can be obtained from pineapple field waste, which is abundantly available in Thailand [26,27,28]. We reported that the PSS film exhibits good properties, such as high water resistance, low water absorption, and good mechanical strength, while still being readily biodegradable [29]. As a result, the PSS film has been proposed for use in single-use or disposable applications.

This study aims to extend the potential applications of PSS by developing a biodegradable composite suitable for single-use purposes. The high amylose content of PSS is expected to confer good water-resistant properties to the composite. The matrix material used for the composite was unmodified and raw PSS. The mechanical properties of the material were enhanced by adding glycerol and calcium carbonate as modifiers. By exploring the range of mechanical properties achievable through these modifications, we can identify suitable applications for our composite material. This knowledge is critical for developing sustainable alternatives to conventional plastics and reducing the environmental impact of single-use products.

Glycerol was chosen as the primary plasticizer in this study due to its widespread use and effectiveness in previous research [5,30,31] and its availability as a byproduct from the biodiesel industry [32,33,34]. Similarly, calcium carbonate was chosen as the primary filler due to its wide use in plastic manufacturing and its availability from natural sources, such as the Earth’s crust [35,36], or byproducts from food industries, such as eggshells and seashells [37,38]. Notably, the use of PSS, a nonconventional food-grade starch, in this research ensures that it does not pose a threat to food security. This approach not only makes use of waste or byproducts but also supports the principles of a circular economy and sustainability. The use of renewable resources such as PSS, glycerol, and calcium carbonate in the development of biodegradable composites can significantly reduce reliance on nonrenewable resources and mitigate the environmental impact associated with conventional plastics.

## 2. Materials and Methods

### 2.1. Raw Material and Chemicals

Pineapple stem waste, a byproduct of a proprietary bromelain extraction process, was obtained from Hong Mao Biochemicals Co., Ltd., (Rayong, Thailand). In general, the process involves crushing peeled pineapple stems to disrupt the cell structure and extract liquid by centrifugation [28]. The remaining solid material was dried under the sun for a few days and further ground into a powder using a grinder. The stem powder was collected by sieving (80 mesh) to separate the coarse fibers, cell wall, and other solid contaminants, which constitute about 56% of the whole mass. The powder was used as obtained without further washing. The extractive constituents make up about 15% of the dry powder. The characteristics of the powder are similar to that obtained by the wet milling process reported previously [27]. Commercial-grade glycerol was obtained from local stores and the uncoated-grade calcium carbonate was produced by Surint Omya Chemicals (Kok Toom, Thailand) Co., Ltd., (Lopburi, Thailand).

### 2.2. Preparation of Starch Paste and Composites

A starch paste was prepared by mixing a predetermined amount of PSS, water, and glycerol in a glass beaker. The amount of glycerol was varied at 20, 30, 40, and 50% based on the weight of the PSS. For all formulations, the amount of water was fixed at the same weight as PSS. The mixture was left for at least 60 min at ambient condition before being gelatinized in a household microwave (Toshiba, model ER-G33SC(S), Toshiba Thailand Co., Ltd., Nonthaburi, Thailand) set at 50% of maximum power (1100 W) for 90 s. The gelatinized PSS was left to cool down to room temperature. A predetermined amount of calcium carbonate (0, 20, and 30% wt. of PSS) was then added into the paste on a laboratory 2-roll mill until a homogeneous mixture was obtained. The mixing time was approximately 15 min. After mixing, the mixture was sheeted out to a thickness of approximately 1 mm. The sheet was then dried in a hot-air oven at 80 °C for 6 h. The samples were then left in an ambient environment to gain equilibrium moisture content at least 7 days before any measurements were made. The sample code is represented as GXCaY, where X and Y are the amounts of glycerol and calcium carbonate, respectively.

### 2.3. Characterization of PSS Composites

#### 2.3.1. Fourier-Transform Infrared Spectroscopy (FTIR)

The FTIR spectra of the materials were recorded with a spectrophotometer (Frontier, Perkin Elmer, Waltham, MA, USA) in the attenuated total reflection (ATR) mode using a diamond crystal. The measurements were performed at room temperature over a range of 4000 to 400 cm^−1^ with 16 scans and a resolution of 4 cm^−1^.

#### 2.3.2. X-ray Diffraction (XRD)

The X-ray diffraction patterns of the materials were obtained from a benchtop X-ray powder diffractometer (D2 Phaser, Bruker AXS GmbH, Karlsruhe, Germany) using an X-ray wavelength of 1.54 Å with a step scan of 15 s/point over the 2θ of 5–40 degrees. The percentage of crystallinity of each PSS composite sample was determined using the following equation:

Crystallinity (%) = *A*_c_/(*A*_c_ + *A*_a_) × 100 (1)

where *A*_c_ = the area of crystalline region and *A*_a_ = the area of amorphous region. The peaks belonging to calcium carbonate were excluded from the calculation.

#### 2.3.3. Mechanical Properties

Tensile test: Specimens were punched out from a 1 mm sheet with a cutter (ISO 37 type 2 dumbbell die). Tensile tests were carried out on a universal testing machine (Instron 5569, Instron, High Wycombe, UK) according to ISO 527-3 with a long-travel, contact-type extensometer. A crosshead speed of 50 mm/min was used. The secant modulus at 1% and the tensile strength and elongation at break were obtained as average values from five specimens.

Hardness and density: The hardness of the material was determined according to the durometer method or Shore hardness of ISO 7619-1 (Zwick Model 7206.07, Zwick, Ulm, Germany). The density of the material was determined following Archimedes’ principle with a density kit on a laboratory balance (XS105, Mettler Toledo, Greifensee, Switzerland) according to method A of ISO 1183. The specimen was weighed in air and then weighed when immersed in distilled water using a sinker and wire to hold the specimen completely submerged. The measurement was carried out at 25 °C and the density was calculated using the below equation. The water density for the calculation was set at 1.00 g/cm^3^.

Density = *W*_air_/(*W*_air_ − *W*_water_) (2)

where

*W*_air_ = weight of the sample in air.

*W*_water_ = weight of the sample in water.

#### 2.3.4. Morphology

The fractured surfaces of the specimens obtained from the tensile tests were observed with a scanning electron microscope (SEM) (JSM-IT500, JEOL, Tokyo, Japan). The samples were coated with platinum before the observation.

#### 2.3.5. Water Solubility and Absorption

A piece of specimen was immersed in distilled water for 24 h and its weights (wet and dried) were monitored. The amount of water absorbed was determined following ISO 62 at 25 °C. The water resistance was determined qualitatively by observing the uptake of water by the sheet samples. The water solubility and absorption of the sheets were determined from the following equations:

Water solubility = ((*w*_i_ − *w*_fd_)/*w*_i_) × 100 (3)

Water absorption = ((*w*_f_ − *w*_i_)/*w*_i_) × 100 (4)

where

*w*_fd_ is the weight of the dried PSS sheet after being immersed in distilled water.

*w*_f_ is the weight of the wet PSS sheet after being immersed in distilled water.

*w*_i_ is the initial weight of the PSS sheet.

#### 2.3.6. Soil Burial Test

This test can be used to determine the biodegradability of starch-based materials by microorganisms [39]. The test was slightly modified from a protocol reported previously [39]. Specimens of size 4.0 × 4.0 cm^2^ were cut and put in envelopes made from a high-density polyethylene net for easy recovery. The envelopes were buried at the edge of a garden of the department building, about 10 cm beneath the surface. The pH of the soil was measured to be 7.5. The area was under the shade of trees and was watered every week. No attempt was made to regulate the moisture content and temperature of the area to obtain a natural environment. The envelopes were taken out for the observation of samples after different periods of time. The state of biodegradation was evaluated visually.

### 2.4. Statistical Analysis

Statistical analysis was performed using analysis of variance (ANOVA) with the Data Analysis tool in the Microsoft Excel (Office16) program. The *t*-test method, with two-sample assuming unequal variances, was performed to analyze differences among the means at a confidence level of 95%.

## 3. Results and Discussion

### 3.1. Fourier-Transform Infrared Spectroscopy (FTIR)

**Figure 1:**
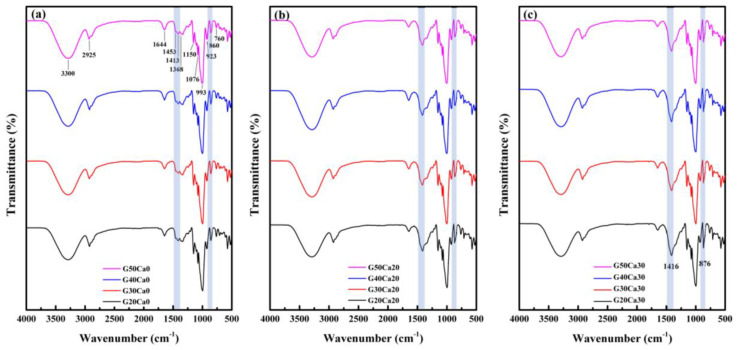
FTIR spectra of PSS composites containing different amounts of glycerol and calcium carbonate: (**a**) no calcium carbonate, (**b**) 20% calcium carbonate, and (**c**) 30% calcium carbonate.

Figure 1 displays the FTIR spectra of the PSS composites containing different amounts of glycerol and calcium carbonate. In the controlled system without calcium carbonate, the amount of glycerol did not significantly affect the FTIR spectra of the composites. The FTIR spectra of the PSS with only glycerol were very similar to that of other starches that have been well understood and documented [40,41]. The peak positions and their corresponding functional group vibrations are listed in Table 1. When calcium carbonate was added, obvious changes were noticed at two positions (shaded area in Figure 1) belonging to calcium carbonate, i.e., the asymmetric stretching peak at 1416 cm^−1^ and out-of-plane bending at 876 cm^−1^ [42]. The intensity of these peaks increased on increasing the amount of calcium carbonate.

### 3.2. X-ray Diffraction (XRD)

**Figure 2:**
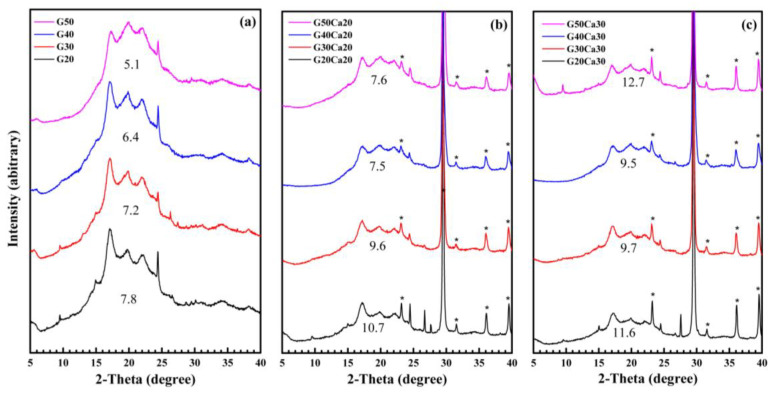
X-ray diffraction patterns of PSS composites containing different amounts of glycerol and calcium carbonate: (**a**) no calcium carbonate, (**b**) 20% calcium carbonate, and (**c**) 30% calcium carbonate. The number on each pattern indicates the crystallinity of the starch matrix. * indicate calcium carbonate diffraction peaks.

Figure 2 displays the XRD patterns of the PSS composites containing different amounts of glycerol and calcium carbonate. All PSS composites have a certain degree of crystallinity. The crystalline structure is different from that of the original PSS, which has an A-type structure, similar to other types of starches as shown previously [27]. The appearance is similar to that of PSS film prepared by solution casting [29] and that of ozonated cassava starch films [11]. These crystalline peaks are attributed to the spontaneous recrystallization of amylose molecules during film drying [43,44] or retrogradation. It was stated that retrograded starch is always B-type regardless of the starch type [11,45]. The crystallinity index decreased on increasing the amount of glycerol added. In systems containing calcium carbonate, five new peaks appeared, which were characteristics of calcium carbonate. The patterns were simply a combination of the PSS matrix and calcium carbonate. The diffraction intensity of PSS dropped significantly but still displayed crystallinity. The drop in intensity is due to the lesser amount of PSS within the measurement volume of XRD. Again, for each set of calcium carbonate contents, the crystallinity index decreased on increasing the amount of glycerol. It is worth noting that most reported thermoplastic starches do not exhibit such a distinct crystalline structure as is observed here [20,25,46]. This is presumably due to the high amylose content of PSS as it is the component that undergoes rapid reordering to form double helices and crystallites [47,48].

### 3.3. Mechanical Properties

**Figure 3:**
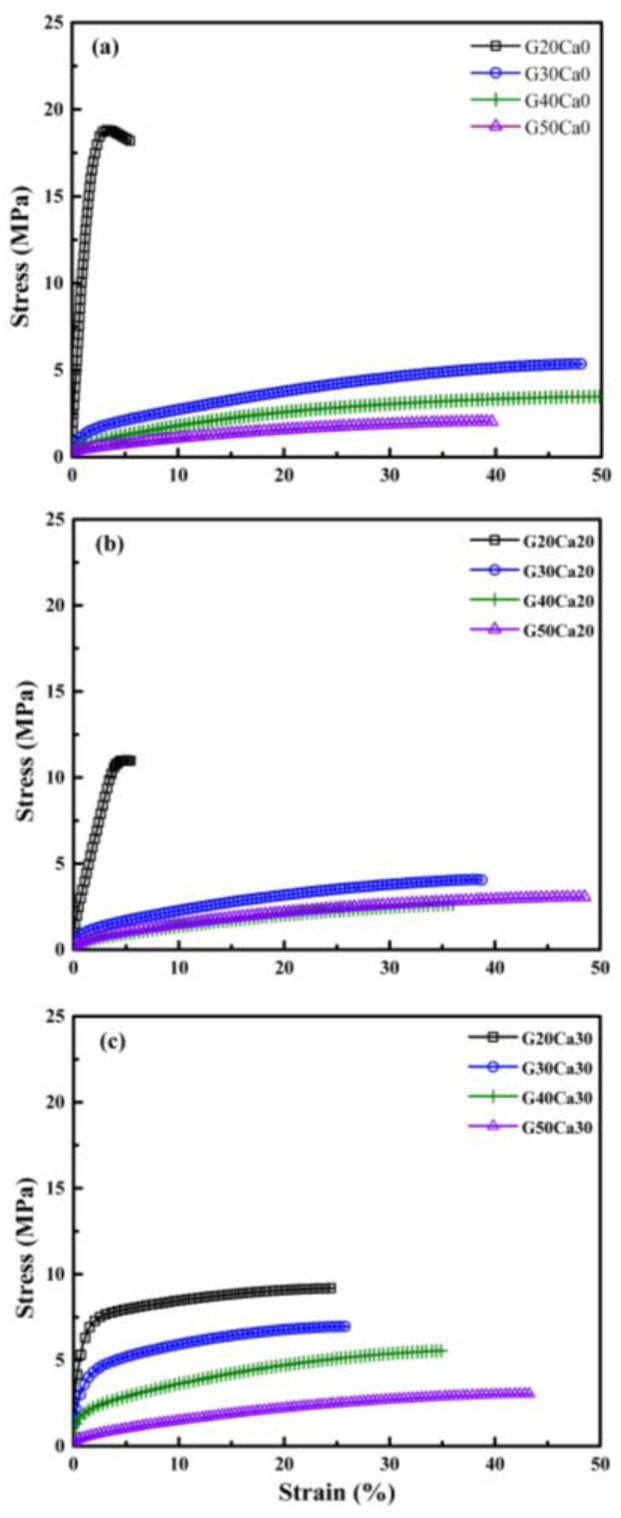
Stress–strain curves of PSS composites containing different amounts of glycerol and calcium carbonate: (**a**) no calcium carbonate, (**b**) 20% calcium carbonate, and (**c**) 30% calcium carbonate.

**Figure 4:**
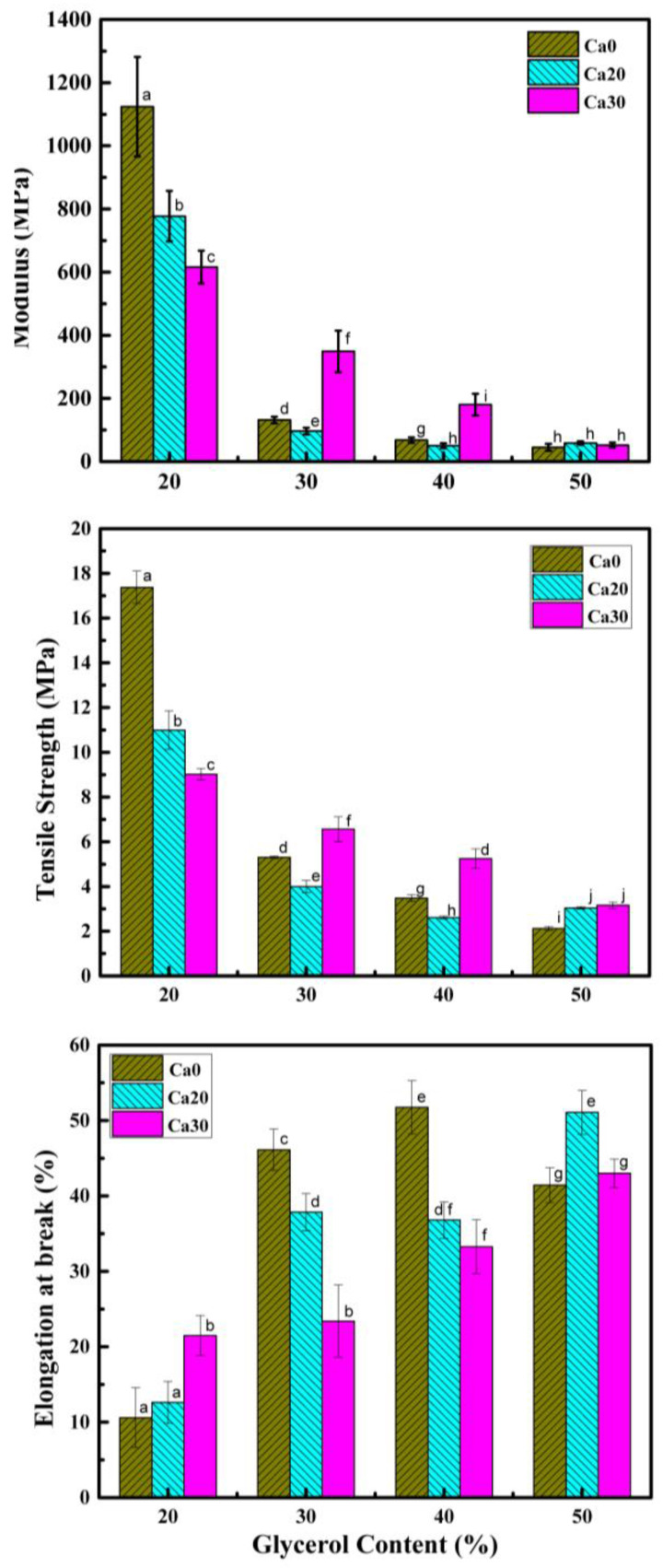
Modulus, tensile strength, and elongation at break of PSS composites containing different amounts of glycerol and calcium carbonate. Different letters on each bar indicate statistically significant differences in the means.

Figure 3 displays the stress–strain curves of the PSS composites containing different amounts of glycerol and calcium carbonate. For the system without calcium carbonate (Ca0), that with 20% glycerol displayed a sharp rise in stress as it was extended, and the stress reached a maximum point and then dropped slightly and broke at about 5% strain. When the glycerol content increased to 30, 40, and 50%, the stress dropped significantly, but the material could still be extended to a strain of about 50%. The maximum stress decreased with increasing glycerol content. When 20% calcium carbonate was added, the stress dropped from that without calcium carbonate but still broke at a strain of about 5%. On increasing the glycerol content to 30% and beyond, a similar pattern was observed, i.e., the stress dropped sharply and further decreased with increasing glycerol content and failure strains increased to about 40–50%. For the last set with 30% calcium carbonate, a very different pattern of behavior was seen. The composite with a glycerol content of 20% displayed a sharp rise in stress and then leveled off and failed at much a greater strain of about 25%. On increasing the glycerol content to 30, 40, and 50%, the stress dropped in steps while the failure strain also increased in steps, reaching a value of about 45%. Average values for the moduli, tensile strength, and elongation at break are shown in Figure 4. The range of moduli obtained was about 45–1120 MPa, and the tensile strength was about 3.0–17.4 MPa. It should be noted that the modulus and tensile strength of the PSS sheet with glycerol content of 20 wt.% were much greater than that of other starches with similar glycerol content, which were about 95.0–529.0 MPa and 5.7–12.0 MPa, respectively [49,50].

**Figure 5:**
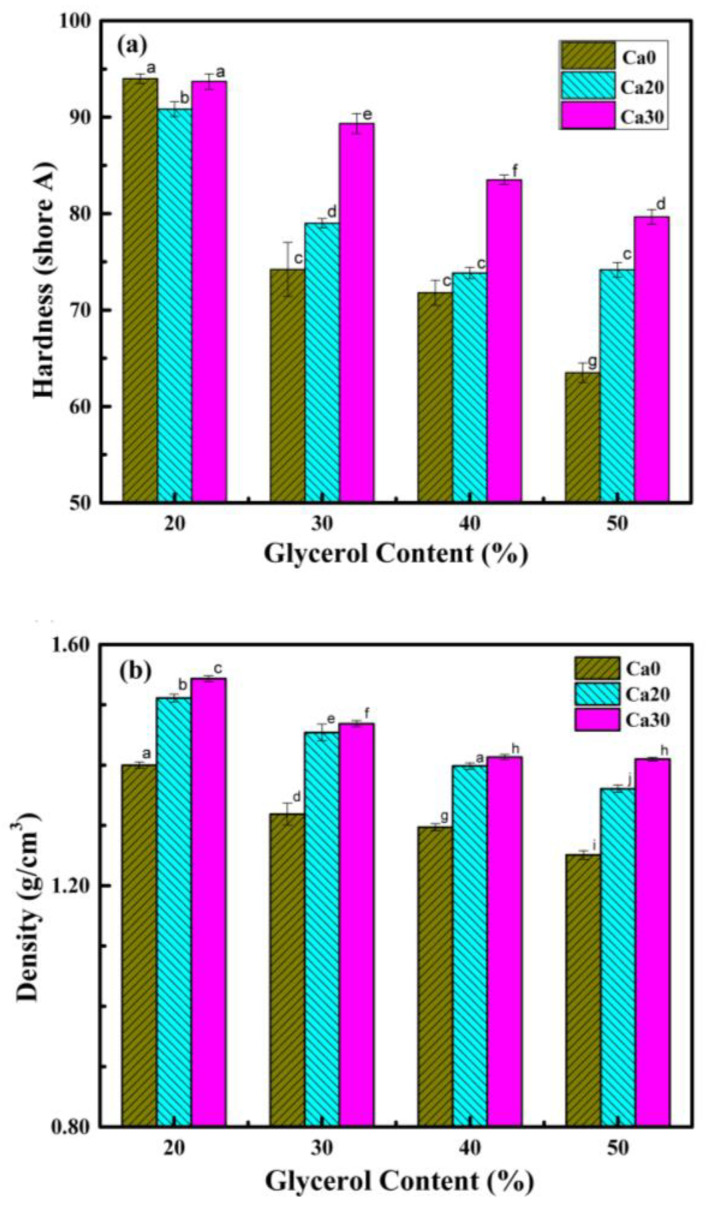
Hardness (**a**) and density (**b**) of PSS composites containing different amounts of glycerol and calcium carbonate. Different letters on each bar indicate statistically significant differences in the means.

Figure 5 displays the hardness of the PSS composites containing different amounts of glycerol and calcium carbonate. At the lowest glycerol content of 20%, the hardness of the composite was about 90–95 Shore A, and the hardness decreased with increasing glycerol content. The lowest hardness obtained was about 63 Shore A for PSS with 50% glycerol without calcium carbonate. For each glycerol content, the hardness increased with increasing calcium carbonate content.

The densities of the PSS composites containing different amounts of glycerol and calcium carbonate are shown in Figure 5b. A trend similar to that for hardness was observed here. For each set of calcium carbonate contents, the density decreased with increasing glycerol content, and for each glycerol content, the density increased with increasing calcium carbonate. These results are to be expected as calcium carbonate has a density of 2.65 g/cm^3^ [35] while that of thermoplastic starch with 35 wt.% glycerol is about 1.4 g/cm^3^ [51]. By assuming these values, the observed densities of the PSS composite sheets fit well with the calculated values.

### 3.4. Morphology

**Figure 6:**
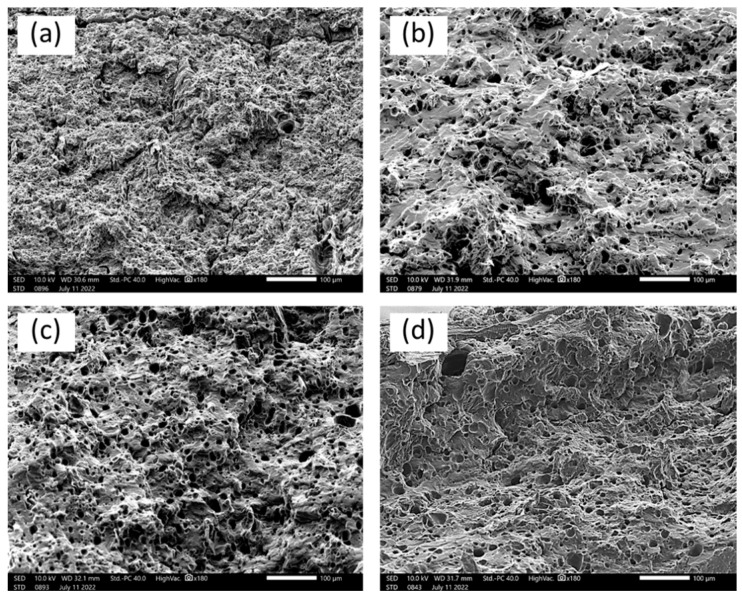
SEM micrographs of tensile-fractured surface of (**a**) G20Ca0, (**b**) G30Ca0, (**c**) G40Ca0, and (**d**) G50Ca0 specimens (scale bar = 100 μm).

**Figure 7:**
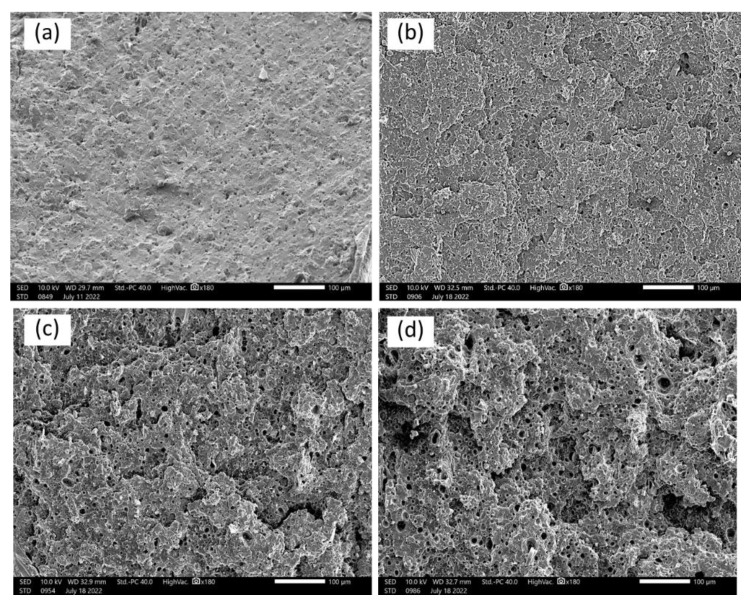
SEM micrographs of the tensile-fractured surface of (**a**) G20Ca20, (**b**) G30Ca20, (**c**) G40Ca20, (**d**) G50Ca20 specimens (scale bar = 100 μm).

**Figure 8:**
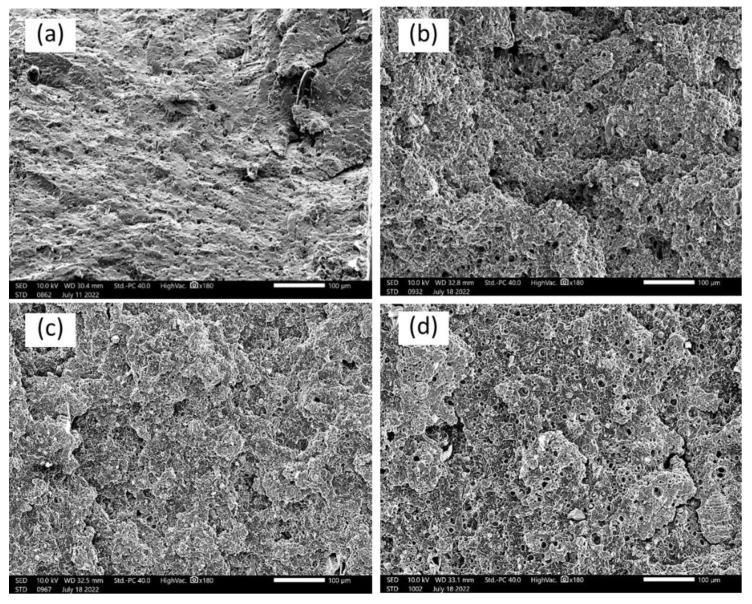
SEM micrographs of the tensile-fractured surface of (**a**) G20Ca30, (**b**) G30Ca30, (**c**) G40Ca30, (**d**) G50Ca30 (scale bar = 100 μm).

Figure 6, Figure 7 and Figure 8 display the scanning electron micrographs of tensile-fractured specimens of G20Ca0, G30Ca0, G40Ca0, and G50Ca0. It is apparent that all specimens contained numerous voids. Presumably these voids occurred because of syneresis process in which water is expelled from the starch network due to retrogradation [52] and then evaporates away in the drying stage. The presence of voids agrees well with the decrease in density with increasing glycerol content observed in Figure 5b. This observation can further support the decrease in tensile modulus and tensile strength of the films with increasing glycerol content. For specimens without calcium carbonate (Figure 6), the fracture surface displayed a very rough morphology. It seems that as the amount of glycerol increases, the size of the voids increases. With calcium carbonate added, the fracture surface showed brittle behavior and no other feature was seen.

### 3.5. Water Solubility and Absorption

**Figure 9:**
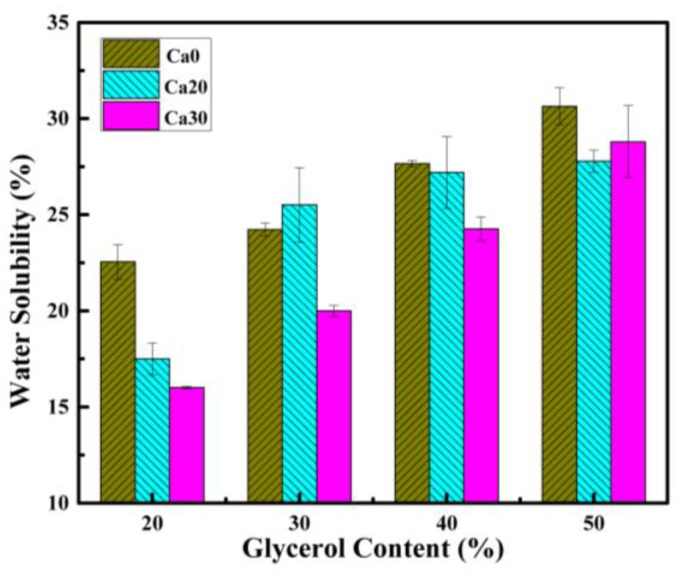
Water solubility of PSS composites containing different amounts of glycerol and calcium carbonate.

Figure 9 displays the water solubility of different PSS composite sheets. Solubility increases with increasing glycerol content indicating more material is leached out. Since both starch and glycerol are water soluble, it follows that the leached material could be both. With the addition of calcium carbonate, the water solubility decreased but still increased with increasing glycerol content. This is to be expected as calcium carbonate is not water soluble. Considering that glycerol molecules are small and readily soluble in water and the water solubility is close to but less than the amount of glycerol added, it is likely that some glycerol could still be trapped inside the composites.

**Figure 10:**
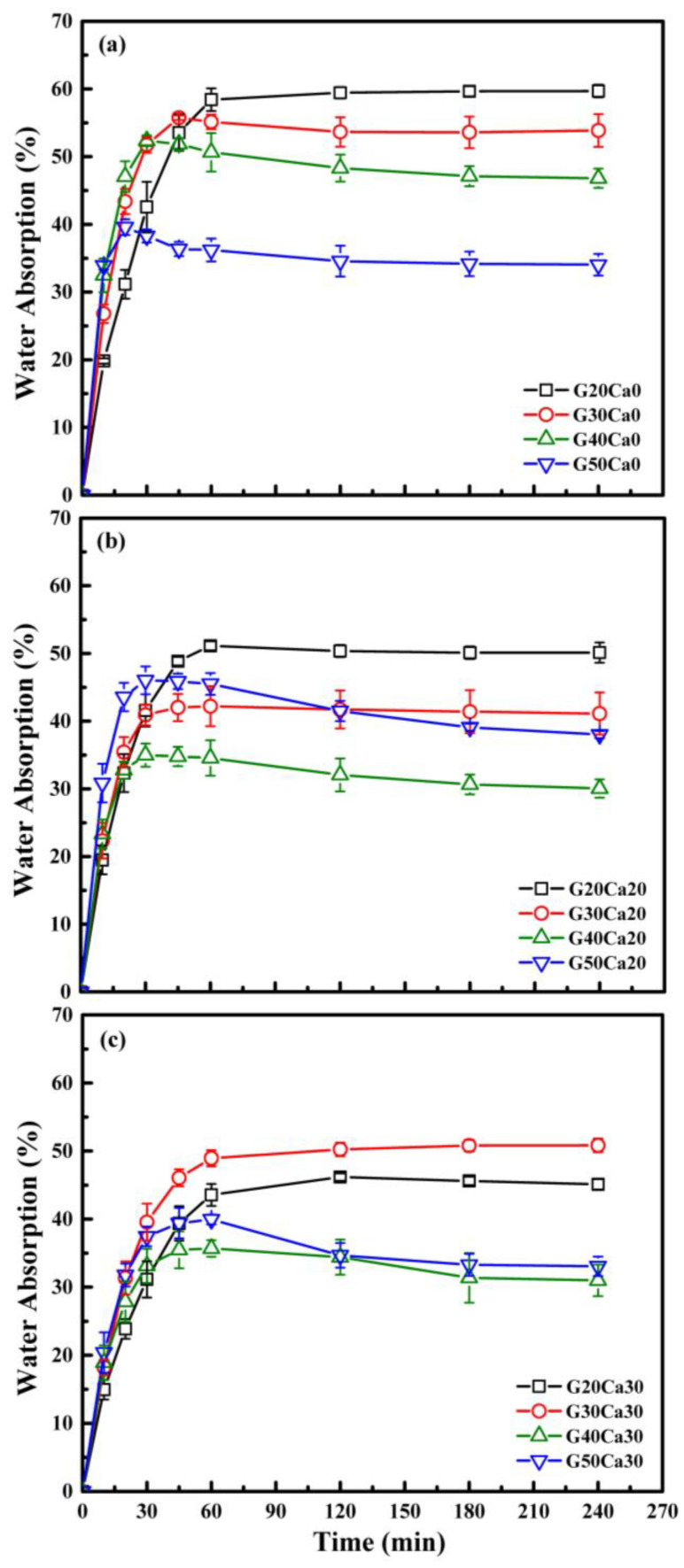
Water absorption of PSS composites containing different amounts of glycerol and calcium carbonate of 0 (**a**), 20 (**b**) and 30 (**c**) wt.%.

Figure 10 displays the water absorption of the PSS composite sheets. For all composites, it is seen that the water absorption increased with increasing immersion time and then leveled off after a certain period of time. The points where the water absorption starts to level off seem to change with the calcium carbonate content, i.e., it increased with increasing calcium carbonate content. In addition, the maximum water absorption for each set of calcium carbonate content depends on the glycerol content. For specimens without calcium carbonate, maximum water absorption decreased with increasing glycerol content. These data should not be treated as evidence for actual lower water absorption since glycerol leaching did occur as will be shown in the next section, and more discussion will follow.

For composites with calcium carbonate, Figure 10b,c, the maximum water absorption decreased, and similar trend was seen that maximum water absorption decreased with increasing glycerol content, but the change was not regularly spaced as in the system without calcium carbonate. This could suggest complex interactions within the structure of the composites. To clearly understand the behavior, further work would be needed, and this will be reported in future correspondence.

### 3.6. Soil Burial Test

**Figure 11:**
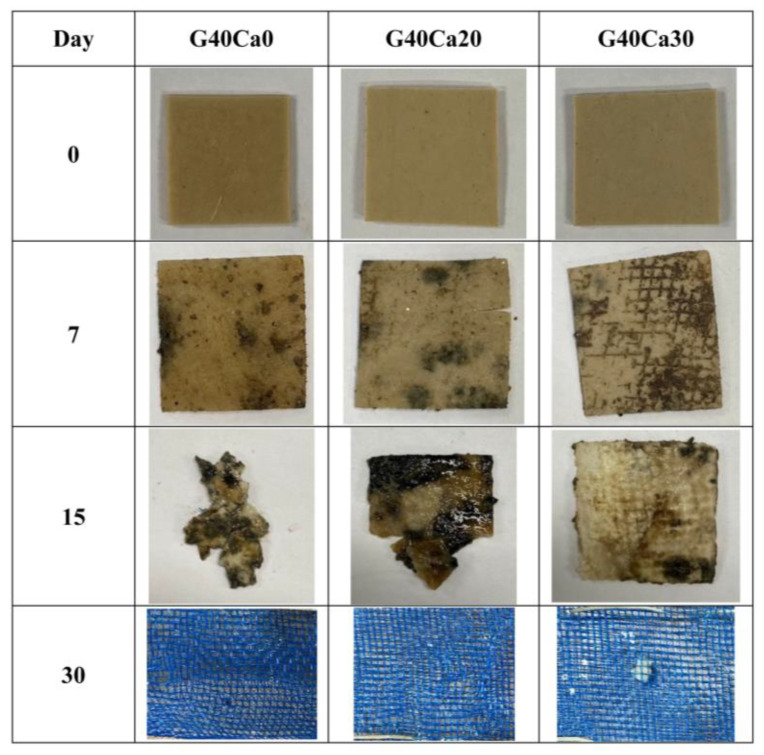
Photographs of some PSS composite specimens before and after burial in the soil for different periods of time.

Figure 11 displays photographs of some selected composite samples before and after the burial test. All composite samples clearly deteriorated in the burial test but at different rates. It appears that, after 7 days, the composites became moldy but still maintained their original shape. After 15 days, composites with little or no CaCO_3_ were broken into small pieces, while those with a higher content largely retained their shape. After 30 days, all composites completely disintegrated, and nothing could be recovered.

## 4. Discussion and Potential Applications

**Figure 12:**
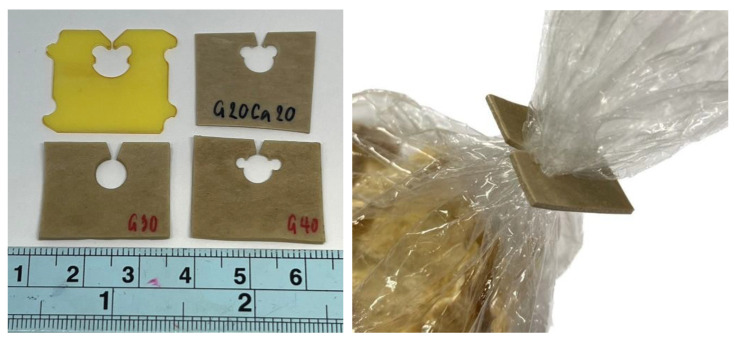
Photographs of simple bread clips made from PSS composites compared with a commercial clip (**left**) and its use for closing a plastic bag (**right**).

It has been shown that composites with a wide range of properties can be prepared from pineapple stem waste, an abundant agricultural waste in Thailand and many other countries. A wide range of properties were obtained from the inherent property of PSS, which has a high amylose content, due to its ability to accept a large amount of plasticizer and filler. The material is completely biodegradable in a natural environment. Thus, properties can be adjusted or tailored to suit different applications. It can be used to replace synthetic plastics in applications where collection for recycling is difficult or not economical and the plastic is likely to leak into the environment, such as bread clips, cotton buds, or golf tees. Compostable versions of these types of products are being offered, such as cardboard bread clips [53]. To demonstrate potential applications of the PSS composite sheet, simple bread clips were cut from a sheet using a manual punching tool, and their photographs are shown in Figure 12. The bread clip was chosen as an example as its function is just to carry some information (related to the product in the packaging) and allow re-closure of a bag with very little stress on the clip. Some composite formulations were found to be too hard and broke during punching, while some gave a good cut. The clips were found to be able to close a sample plastic bag nicely as shown in Figure 12. The clip in the figure was cut from G40Ca0 (without calcium carbonate).

In summary, our study demonstrated the potential of using pineapple stem starch, glycerol, and calcium carbonate for the production of biodegradable composites. The utilization of waste, byproducts, and renewable sources for these materials offers numerous advantages, including a reduction in the energy required for raw material production, lower carbon dioxide emissions, and a reduced need for land and water resources, compared to the use of edible starch. Furthermore, the use of pineapple stem starch, which is not conventionally used as food, does not pose a threat to food security, and edible starches can be reserved for food production. We have also shown that a range of composite formulations can be achieved with varying mechanical and water-resistant properties, making them suitable for various applications. For instance, our proposed application of using the composite as bread clips is a practical example of how it can be used to replace single-use plastics in everyday items that are too small for people to collect for recycling. Moreover, the observed range of properties could serve as a starting point for future research, where other sustainable materials can be incorporated to obtain specific properties tailored to various applications. Overall, our findings hold great potential for advancing sustainable materials and circular economy, reducing plastic waste, and mitigating the environmental impact of plastic production and disposal. With the increasing concern over climate change and resource depletion, our study offers a promising solution toward a more sustainable future.

## 5. Conclusions

Biodegradable plastic sheets with a wide range of mechanical properties were successfully developed from PSS. The high amylose content allows a sufficient degree of crystallinity to impart a good starting point. The use of simple chemicals, such as glycerol as plasticizer and calcium carbonate as reinforcement, provides an opportunity to alter the mechanical properties to suit various applications. The material is specifically useful for applications where strength is not so critical and collection back for recycling is difficult. Since it is starch-based, the material is readily biodegradable within a short period of time and should leave no microplastics and other contaminants behind. In addition, the rate at which the composite deteriorates can be controlled via the filler or other additive contents.

## Figures and Tables

**Table 1:** FTIR peak positions and corresponding functional group vibrations.

No.	Observed Position (cm^−1^)	Functional Group
1	3300	O-H stretching
2	2925	C-H stretching
3	1644	C-O bending (associate with OH group)
4	1453	CH_2_ symmetric deformation
5	1413	CH_2_ symmetric scissoring
6	1368	C-H symmetric bending
7	1150	C-O-C asymmetric stretching
8	1076, 993	C-O stretching
9	923, 860, 760	C-O-C ring vibration of carbohydrate

## Data Availability

The data presented in this study are available on request from the corresponding author.
